# When Familiar Faces Feel Better: A Framework for Social Neurocognitive Aging in a Rat Model

**DOI:** 10.1523/ENEURO.0422-25.2025

**Published:** 2026-02-04

**Authors:** Subhadeep Dutta Gupta, Jeffrey M. Long, Peter R. Rapp

**Affiliations:** Laboratory of Behavioral Neuroscience, National Institute on Aging, Baltimore, Maryland 21224

**Keywords:** cognitive aging, intermittent theta burst stimulation, memory, social cognition, transcranial magnetic stimulation

## Abstract

Social cognition, central to emotional and cognitive well-being, is particularly vulnerable to aging, where impairments can lead to isolation and functional decline. Despite compelling evidence that altered social behavior is associated with cognitive decline and dementia risk, experimental strategies for testing causative links remain scarce. To address this gap, we aimed to establish a rat model for research on social neurocognitive aging. We conducted a large-scale behavioral study in 169 male young (6 months) and aged (24–25 months) Long–Evans rats. In order to explore potential relationships among aging outcomes, we first documented individual differences in a widely validated water maze test of hippocampal learning and memory. Sociability and social novelty were then evaluated in the same subjects using the three-chamber social interaction test. Aging induced a selective shift in social novelty preference, marked by a striking familiarity bias in a substantial subpopulation of old rats, while sociability remained entirely normal. Changes in social novelty preference were completely independent of individual differences in spatial memory and unrelated to anxiety or sensorimotor function. Notably, neuromodulation via TMS enhanced social novelty preference selectively in aged rats that exhibited a social introversion phenotype before treatment, consistent with the possibility that this aging condition reflects a distinct and modifiable neural network state. Together, the results establish a valuable preclinical framework for developing a comprehensive neurobiology of social cognition in aging.

## Significance Statement

Social behavior is a critical yet underexplored component of cognitive aging. While both human and animal studies report age-related narrowing of social networks, the behavioral and neurobiological underpinnings remain unclear. Using a well-powered rat model, here we demonstrate preserved sociability in aging alongside marked individual differences in social novelty preference. A subset of aged rats preferred familiar over novel conspecifics, resembling patterns observed in older humans and nonhuman primates. Social phenotypes were independent of hippocampal-dependent memory, suggesting a dissociation between these aging outcomes. This dissociation was further validated using transcranial magnetic stimulation, supporting the notion of distinct underlying neurobiological mechanisms. Collectively, the findings lay a powerful foundation for advancing the translational neurobiology of social behavior in cognitive aging and reserve.

## Introduction

Humans are living longer, and global demographic shifts predict an increasingly aged population. Inevitable and inherently complex, aging is associated with a multitude of physiological, cognitive, and emotional changes. Among the most affected, social cognition profoundly impacts the quality of life (Frith and Frith, 2007). The recent COVID-19 pandemic was a potent example, highlighting the powerful influence of social connectedness on cognitive, emotional, and physical well-being, with social restrictions disproportionately affecting the aged population (Lebrasseur et al., 2021). Despite growing evidence linking social changes to neurocognitive aging (Ebner et al., 2024; Chander et al., 2025), the field lacks validated preclinical animal models for research into underlying neurobiological mechanisms.

An individual's social architecture is a crucial determinant of overall health and well-being, and increasing evidence points to social isolation as a risk for cognitive decline and dementia (Fratiglioni et al., 2000; Samtani et al., 2022; Sommerlad et al., 2023). Interestingly, motivational priorities tend to shift during aging toward goals from which individuals derive emotional meaning, leading to a familiarity bias and reduced social spheres (English and Carstensen, 2014). Balancing the potential positive benefit on emotional well-being, whether age-related social narrowing is a consequence or driver of broader cognitive decline in later life remains unclear. This shift can also be self-reinforcing, leading to greater reliance on familiar relationships and caregivers (Pinquart and Sorensen, 2006) and limiting adaptability and social flexibility. In contrast, exploring new social circles is cognitively stimulating and promotes overall reserve against dementia (Cai, 2022; Sommerlad et al., 2023), encouraging the view that interventions aimed at enriching social interactions might support healthy aging.

Investigating mechanisms of age-related change in social cognition in long-lived humans and nonhuman primates (NHP) is difficult on practical grounds and complicated by individual personality traits, demographic variability, and cultural factors (Sharifian et al., 2022; Schroeder et al., 2024). Rodent models are a powerful alternative for addressing appropriately targeted aims, allowing strict experimental control and tractable lifespans on the order of a few years. Indeed, a well-validated rodent model is arguably essential for charting the progression of shifts in social dynamics over the life course in relation to other features and underlying mechanisms of neurocognitive aging.

The present study aimed to fill important knowledge gaps in the social neuroscience of cognitive aging by building on a rat model with established value in defining the neurobiological basis of individual differences in hippocampal memory function. Intended as a foundational anchor investigation, we examined multiple social cognitive domains in an unusually large sample of over 150 young and aged rats. The findings indicate that distinct dimensions of social cognition are differently vulnerable to aging, independent of change in olfactory function or temperament. Like many other aging processes, between-subject variability was substantially increased in aged rats, but effects on social cognition were entirely uncoupled from individual differences in memory function. Prompted by our earlier work showing that the effects of transcranial magnetic stimulation (TMS) on recognition memory in aged rats vary based on hippocampal function (Weiler et al., 2021), here we adopted a parallel strategy to test the influence of individual differences in social phenotype on the response to TMS. Together, the results establish a powerful foundation for forging new ground in the translational neurobiology of social influences on cognitive aging and reserve.

## Methods and Materials

### Animals and experimental design

Young adults (*N* = 60; 6 months) and aged (*N* = 109; 24–25 months) male Long–Evans rats (Charles River Laboratories) were singly housed on a 12 h light/dark schedule (lights on at 6.30 A.M.) at the National Institute on Aging (NIA). The aged rats were acquired as retired breeders at 8–9 months of age and were individually housed to prevent fighting and injury, constituting an approved exemption from social housing. Standard rat chow and water were provided *ad libitum*. All procedures were approved by the Institutional Animal Care and Use Committee of the NIA, per the National Research Council Guide for the Care and Use of Laboratory Animals. Daily health checks were conducted, and animals in poor physical or clinical conditions were excluded (Crawley, 2007).

**Figure 1.**
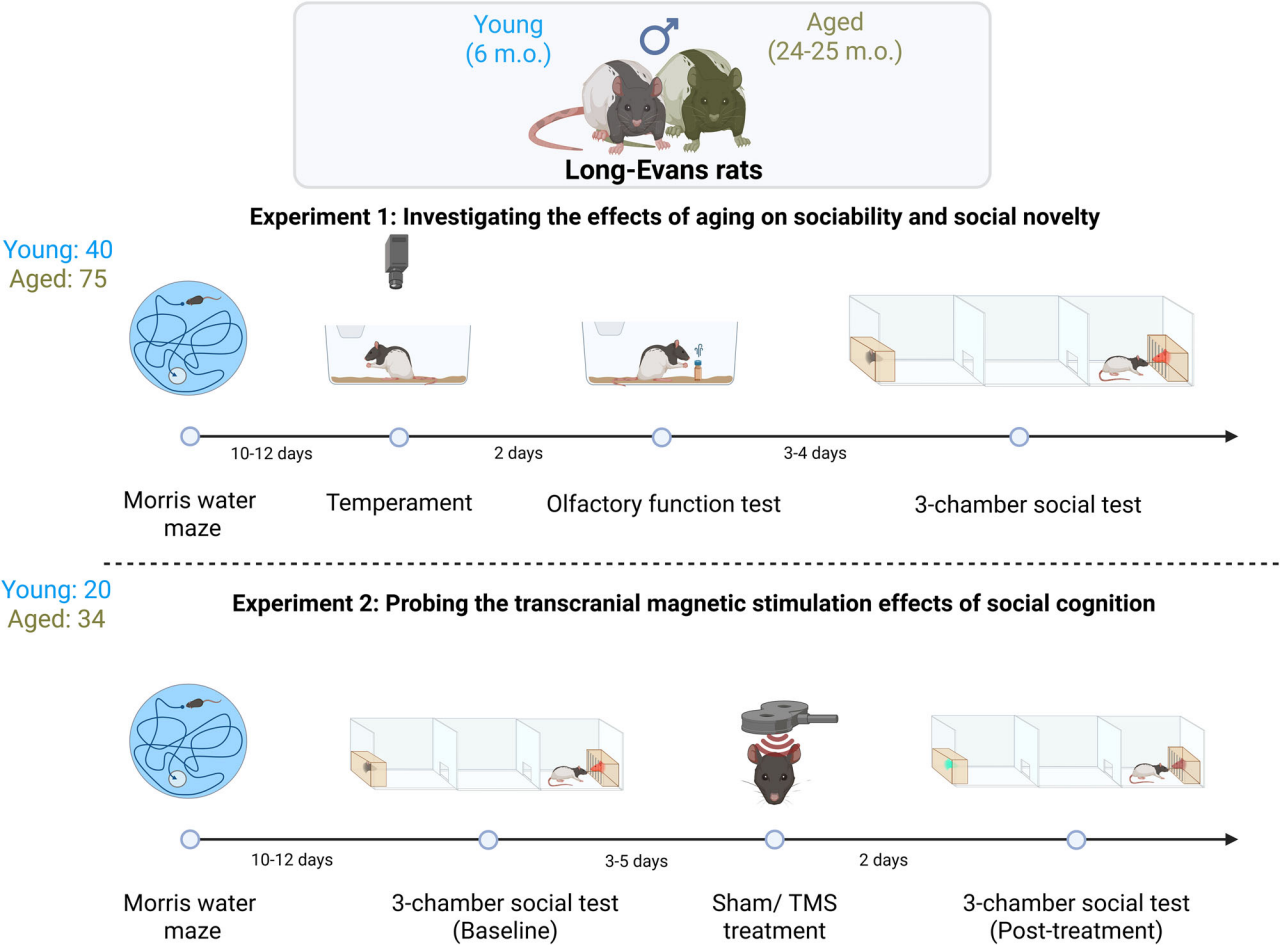
Experimental overview. Sociability and social novelty were tested in young and aged male Long–Evans rats (Experiment 1). A different cohort of rats was used to probe the effects of TMS on social cognition (Experiment 2).

All subjects in the current study were behaviorally characterized in the Morris water maze before assignment to experiments examining aging effects on sociability and social novelty (Experiment 1) or the social novelty response following TMS (Experiment 2). Of the rats tested on the three-chamber procedure (40 young; 75 aged) in Experiment 1, separate subsets also underwent temperament profiling (8 young; 12 aged) or a test of olfactory function (12 young; 26 aged). In Experiment 2, performance on the three-chamber social test was assessed in 20 young and 34 aged rats and in response to sham or TMS treatment. The experimental overview is depicted below (Fig. 1).

### Behavioral testing procedures

#### Morris water maze

Spatial learning and memory were assessed using an extensively validated hippocampus-dependent “place” version of the Morris water maze (Gallagher et al., 1993; Tarbox et al., 2025). Briefly, animals received 8 consecutive days of sparse training (three trials per day) interspersed with four probe trials across days, one on the last trial every other day. During the probe trials, the platform was inaccessible for the first 30 s of the trial, and spatial preference for the escape platform was analyzed (Gallagher et al., 1993). A learning index (LI) score calculated for each animal reflected their proximity (in centimeter) to the hidden escape platform across interspersed probe trials during training (Gallagher et al., 1993). Lower scores indicate greater search accuracy focused on the escape platform, and aged animals scoring > 240 were classified as aged-impaired; those ≤ 240 were considered aged-unimpaired (Gallagher et al., 2011; Koh et al., 2022). A nonspatial, cued version of the task was used to exclude rats with nonmnemonic sensorimotor or motivational deficits.

#### Three-chamber social interaction test of sociability and social novelty

The three-chamber social interaction test was conducted 10–12 d after the water maze to evaluate sociability and social novelty (Moy et al., 2004). Animals were briefly acclimated to a novel test room before being placed in a three-chamber apparatus for social behavioral testing. The custom apparatus (Maze Engineers) featured three adjacent compartments (40 × 80 × 40 cm; L × W × H) divided by walls with 20 × 18 cm openings to allow free exploration throughout the apparatus. Rats were habituated to the empty arena for 10 min. To evaluate general social interest, on the sociability trial, we positioned an identical small enclosure (38 × 20 × 20 cm; L × W × H) in each of the two lateral chambers, one empty and the other containing a naive young or aged male conspecific (including both ages as stimulus rats, allowing assessment of potential “own age” bias in the results). In the social novelty trial that followed immediately, the now-familiar trapped rat from the sociability trial was presented, now paired with a novel conspecific in the previously empty chamber. The arena and enclosures were cleaned between test subjects with 70% alcohol to remove residual odors. The time spent exploring each enclosure was recorded using the ANY-maze software (Stoelting), with zones of interest defined around each stimulus cage. Investigation of either the social or nonsocial stimulus was considered when the experimental animal was within ∼3 cm of an enclosure with its nose oriented toward the front opening. Scoring was validated by an experimenter blind to group identity.

#### Temperament profile

Temperament profiles captured multiple behaviors (grooming, digging, rearing, walking, sniffing, freezing, and no activity) in a random subset of rats held singly in a fresh cage in a novel, dimly lit (70 lux) environment for 10 min (Fuzesi et al., 2016). The cage was placed on a table, and the lid was removed to increase arousal. Behavior was video-recorded and analyzed by an experimenter blind to group identity using a software annotator.

An integrated anxiety *z*-score was calculated using three parameters: rearing and digging during the temperament test and distance traveled in the three-chamber arena:

$$\mathrm{Integrated}\;\mathrm{Anxiety}\;Z\text{-}\mathrm{score}\;=\frac{z\;\mathrm{score}\;(\mathrm{rearing})+\;z\;\mathrm{score}\;(\mathrm{digging})+\;z\;\mathrm{score}\;\mathrm{distance}\;\mathrm{traveled}}{\mathrm{Number}\;\mathrm{of}\;\;\mathrm{parameters}\;(3)},$$

where the *z-*score for individual tests was computed for the young and aged groups separately (Guilloux et al., 2011). To facilitate interpretation, the polarity of the scores was aligned so that both lower rearing and distance traveled, and greater digging reflected higher anxiety-like behavior.

#### Olfactory habituation–dishabituation test

The odor habituation–dishabituation test assessed whether olfactory deficits contribute to age-related change observed in social behavior. On test days, rats were brought into a quiet, dimly lit room and acclimated for 10 min in their home cage. Rats were placed individually in a test cage containing a thin layer of clean corn cob bedding and allowed to habituate for an additional 10 min. Rats were presented with a series of individual odorants—water, lime, almond (McCormick; 200 μl in 1 ml deionized water), and rat urine from a stranger, young male Long–Evans rat, each for three 2 min trials, with 2 min intertrial intervals (Yang and Crawley, 2009). The rat urine served as a social odor and was prepared immediately before each trial. Dishabituation comprised increased vial sniffing when a new odorant was introduced. Habituation was defined as a progressive decrease in sniffing time across repeated presentations of the same odor. A fresh test cage and odor vials were used for each animal. Odor-directed exploration comprised the cumulative time the rat's snout was oriented toward and within 2 cm of the vial opening. Contact with other body parts or climbing on top of the vial were not considered odor-directed exploration. Behavior was video-recorded, and scoring was conducted blind to the group and odor identity.

### TMS

Three to five days following the baseline social cognition test, young and aged rats in Experiment 2 randomly received TMS or sham stimulation as described previously (Weiler et al., 2021, 2023). To minimize stress and head movement during this treatment, rats were initially lightly anesthetized with 1.5% isoflurane and subsequently maintained on dexmedetomidine hydrochloride (aged, 0.03 mg/kg body weight; young, 0.035 mg/kg body weight, i.m.), using a protocol that preserves BOLD signal for resting-state MRI analysis in rats (Lu et al., 2012). Body temperature was monitored (STARR Life Sciences MouseOx Plus) and held stable with a heating pad. Intermittent theta burst stimulation (iTBS) was delivered using a Magstim Rapid^2^ stimulator with a 70 mm figure-eight coil (Magstim Company, RRID:SCR_026437). The coil was centered dorsomedially between the eyes and ears for active stimulation, with the handle perpendicular to the body axis. In the sham condition, the coil was placed 30 cm above the animal's head, facing upward, replicating the auditory effects of iTBS. Stimulation was delivered in three bouts, spaced 3 min apart, each lasting 192 s during a 15 min session. iTBS bouts comprised 20 trains of three bursts of 50 Hz pulses repeated at 5 Hz for 2 s, with a 10 s interval between trains. Each 192 s bout delivered 600 pulses, resulting in 1,800 pulses cumulatively across the entire session. Stimulation intensity was set at 15% of the maximum output based on evoked motor responses in four anesthetized pilot rats. The effects of dexmedetomidine were reversed by atipamezole hydrochloride injection (young, 0.35 mg/kg body weight; aged, 0.3 mg/kg body weight, i.m.) immediately following stimulation or sham. All animals recovered within ∼2 min. Posttreatment social behavior was assessed using a different set of trapped conspecifics in the three-chamber test 2 d after the administration of either sham or TMS.

### Statistical analysis

Absent established background data for an empirically informed power analysis, we instead opted for an unusually large sample size to ensure sensitivity and reproducibility. Exclusion criteria were failure on the nonspatial, cued variant of the water maze, failure to sample all available choices in the social and olfactory tests, or the presence of pituitary tumors detected postmortem. Normality was examined using the D'Agostino and Pearson's omnibus test. Age contrasts (young vs aged) were tested by an unpaired Student's *t* test. Multivariate analyses were conducted using one-way and two-way ANOVA, with post hoc tests as appropriate. Potential bivariate associations were tested using Pearson's correlations. The effect of sham or TMS treatments was analyzed by two-way repeated–measure (RM) ANOVA, with pre- versus posttreatment compared by paired *t* tests, considering each group separately. *P* values <0.05 were considered statistically significant, and trends were noted for values between 0.05 and 0.1. All statistical analyses were performed using GraphPad Prism 10.0.2 (RRID: SCR_002798).

## Results

### Aged rats show preserved sociability but a familiarity bias with increased interindividual variability

**Figure 2.**
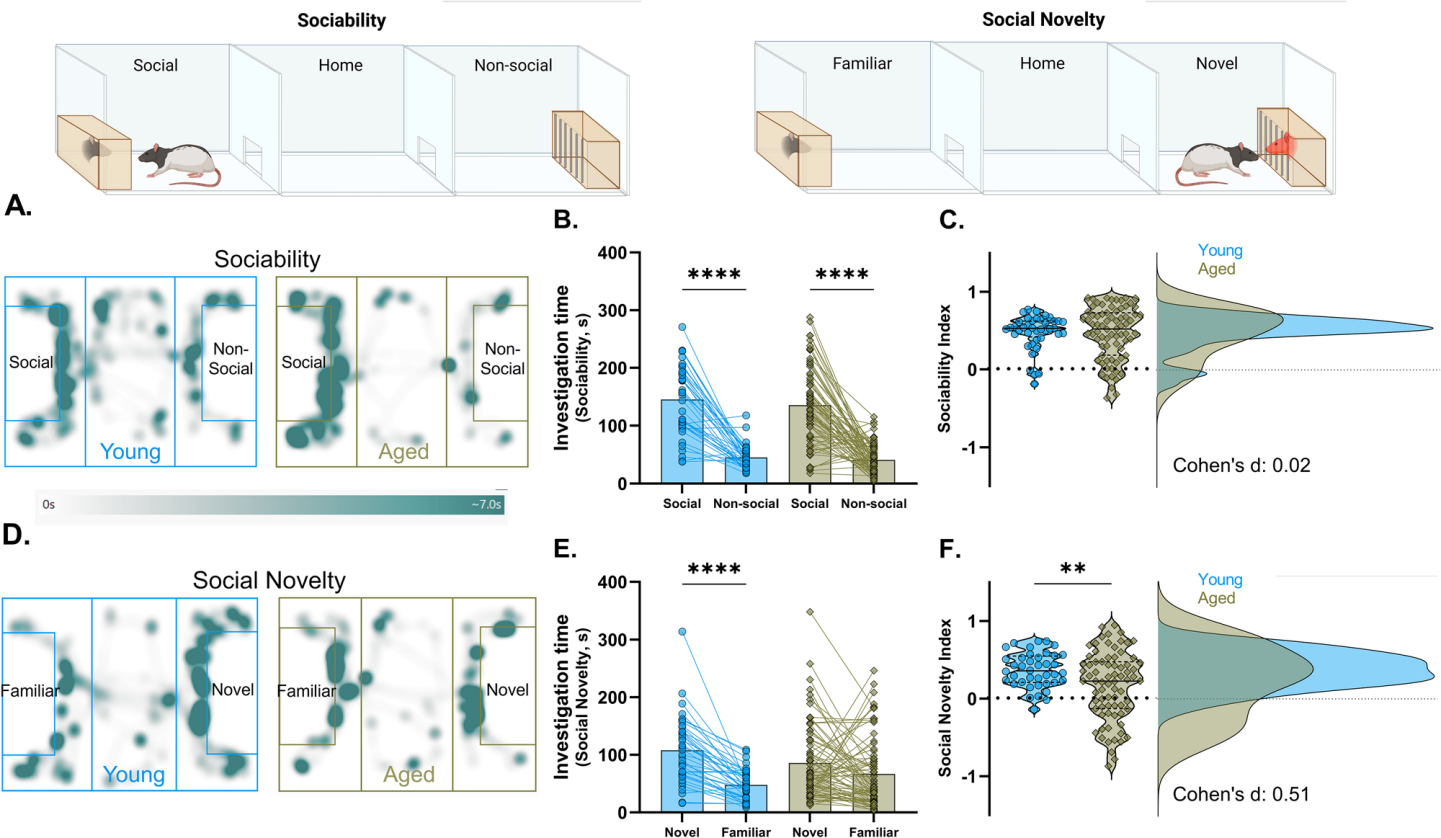
Aged rats were social but showed a decreased preference for social novelty in the three-chamber social test. ***A***, Heat maps showing the distribution of exploration in representative young and aged rats on the sociability trial. ***B***, Mean and individual animal social and nonsocial investigation times on the sociability trial. Two-way ANOVA: main stimuli effect, *F*_(1,113)_ = 201.3; *p* < 0.0001. Bonferroni's multiple-comparison test, ****p < 0.0001. ***C***, Violin plot of the sociability index scores (left) and frequency distribution of the scores (right) for young and aged rats. ***D***, Heat maps depicting exploration patterns in representative young and aged rats during the social novelty trial. ***E***, Mean and individual subject novel and familiar investigation times on the social novelty trial. Two-way ANOVA: main novelty effect, *F*_(1,113)_ = 31; *p* < 0.0001; novelty × age interaction, *F*_(1,113)_ = 8.6; *p* = 0.004; ****p < 0.0001. Bonferroni's multiple-comparison test, ****p < 0.0001. ***F***, Violin plot of the social novelty index scores (left, unpaired *t* test, *t*_(113)_ = 2.62; **p = 0.009) and frequency distribution for the young and aged groups (right; effect size = 0.51; unpaired Cohen's *d*, 95% CI, −0.83 to −0.2). *F* test for interindividual variability (*F*_(74,39)_ = 3.9; *p* < 0.0001). *n*, young = 40; aged = 75 rats. See Extended Data Figures 2-1 and 2-2 for own age bias, locomotor activity, and total stimulus-driven exploration of the experimental animals in the three-chamber social test.

To understand the influence of aging on social cognition, we investigated sociability and social novelty in young and aged rats using the three-chamber social interaction test. Young and aged rats displayed a strong and equivalent preference (Fig. 2*A*,*B*; Table 1) for investigating an unfamiliar rat over a nonsocial object, indicating a substantial degree of sociability in both groups (Fig. 2*C*; Table 1). On the immediately following social novelty trial, young rats spent significantly more time with the novel conspecific than the now-familiar rat encountered during the sociability test (Fig. 2*D*,*E*). Whether the novel rat was young or aged had no effect; “own age” bias was not observed in any group (Extended Data Fig. 2-1). However, results for the aged rats were strikingly different from young in two ways. First, as a group, aged rats failed to display a reliable social novelty preference overall (Fig. 2*D*,*E*; Table 1), and on average they scored lower than young (Fig. 2*F*). Second, interindividual variability was significantly greater among old animals, with nearly half exhibiting a phenotype not seen in the young group, comprising an apparent social bias for the familiar conspecific (Fig. 2*F*).

10.1523/ENEURO.0422-25.2025.f2-1 Fig 2-1 **Own age bias in young and aged experimental rats.** (A) Violin plots with median showing the preference of young and aged rats toward young and aged trapped (stimulus rats) in sociability (A) and social novelty (B) trials. Both age groups showed a similar magnitude of preference for either a young (4-6 months old) or an aged (24-25 months old) trapped conspecific in these trials. The sample size of each condition is depicted below the violin plots. Download Fig 2-1, TIF file.

10.1523/ENEURO.0422-25.2025.f2-2 Fig 2-2 **Locomotor activity and stimulus-driven exploration of young and aged rats.** (A) Box and whisker plots depicting the distance traveled per subject for each phase of testing. Two-way ANOVA: main effect of group: *F*_1,113_ = 39.06; *p* < 0.0001, stimuli or novelty: *F*_2,226_ = 46.32; *p* < 0.0001, and their interaction: *F*_2,226_ = 4.89; *p* = 0.008. Bonferroni’s multiple comparisons test, **p < 0.01, ****p < 0.0001. (B) Box and whisker plots depicting total stimulus-directed exploration time, with individual animal values. S: Social, N.S: Non-social, N: Novel, F: Familiar. Two-way ANOVA: main effects of group: *F*_1,113_ = 3.58; *p* = 0.52, trials: *F*_1,113_ = 0.4; *p* = 0.53, and their interaction: *F*_1,113_ = 0.76; *p* = 0.39. *n*: young = 40, aged = 75 rats. Download Fig 2-2, TIF file.

**Table 1.** Descriptive statistics for the three-chamber social test metrics (mean ± SEM)

	Parameters	Young	Aged
Habituation	Distance traveled (meters)	25.38 ± 1.25	21.08 ± 1.01
	Chamber 1 time (seconds)	163.7 ± 11.26	198.36 ± 16.54
	Home time (seconds)	252.78 ± 13.75	205.98 ± 15.41
	Chamber 2 time (seconds)	185.7 ± 11.32	195.62 ± 14.82
Sociability	Time social (seconds)	145.3 ± 9.57	135.7 ± 7.53
	Time nonsocial (seconds)	45.42 ± 3.21	41.07 ± 2.99
	Distance traveled (meters)	26.36 ± 1.25	17.83 ± 0.71
Social novelty	Time novel (seconds)	107.9 ± 9.11	85.8 ± 7.51
	Time familiar (seconds)	47.79 ± 4.13	66.92 ± 6.92
	Distance traveled (meters)	20.58 ± 1.32	12.4 ± 0.56

### Social cognitive functions are dissociated from spatial learning and memory

**Figure 3.**
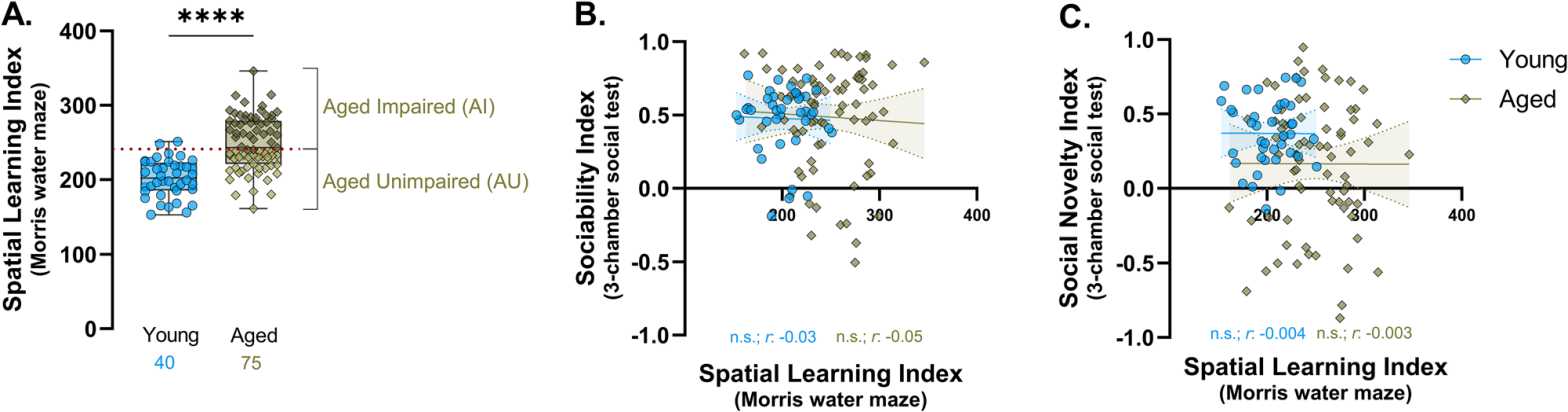
Individual differences in spatial learning and memory are independent of social novelty preference. ***A***, Median box and whisker plot with distribution of LI scores for young and aged rats. The red-dotted line represents the cutoff (240) for classifying aged rats as impaired (dark shade) and unimpaired (light shade). Unpaired *t* test, *t*_(113)_ = 7.35; ****p < 0.0001. ***B***, ***C***, Bivariate scatterplots of sociability (Pearson's correlation, *r*, young = −0.03; aged = −0.05) and social novelty (Pearson's correlation, *r*, young = −0.004; aged = −0.003) indices with water maze LI scores. Data from aged-impaired and unimpaired rats are pooled in the “aged” group. *N*, young = 40; aged = 75 rats.

The social novelty component of the three-chamber procedure is operationally analogous to a recognition memory test, with a short retention interval and minimal memory demand. By this view, it might be that the effects of aging on hippocampal memory account for observed individual differences in social novelty rather than a change in social cognition, per se. Consistent with many earlier studies (Myrum et al., 2019; Chen et al., 2024; Cooper et al., 2024), as a group, aged rats in the current experiment displayed significant spatial memory deficits relative to the young (Fig. 3*A*), together with substantially greater interindividual variability in LI scores. However, a direct analysis of results from our sample of 40 young and 75 aged rats found that both sociability (Fig. 3*B*) and social novelty (Fig. 3*C*) are entirely uncoupled from variability in spatial memory. The risk of false negatives in such large groups is small, and the results instead suggest that changes in social novelty and hippocampal memory are independent features of cognitive aging in this model.

### Age-related shift in social novelty preference is not attributable to anxiety or sensorimotor functions

**Figure 4.**
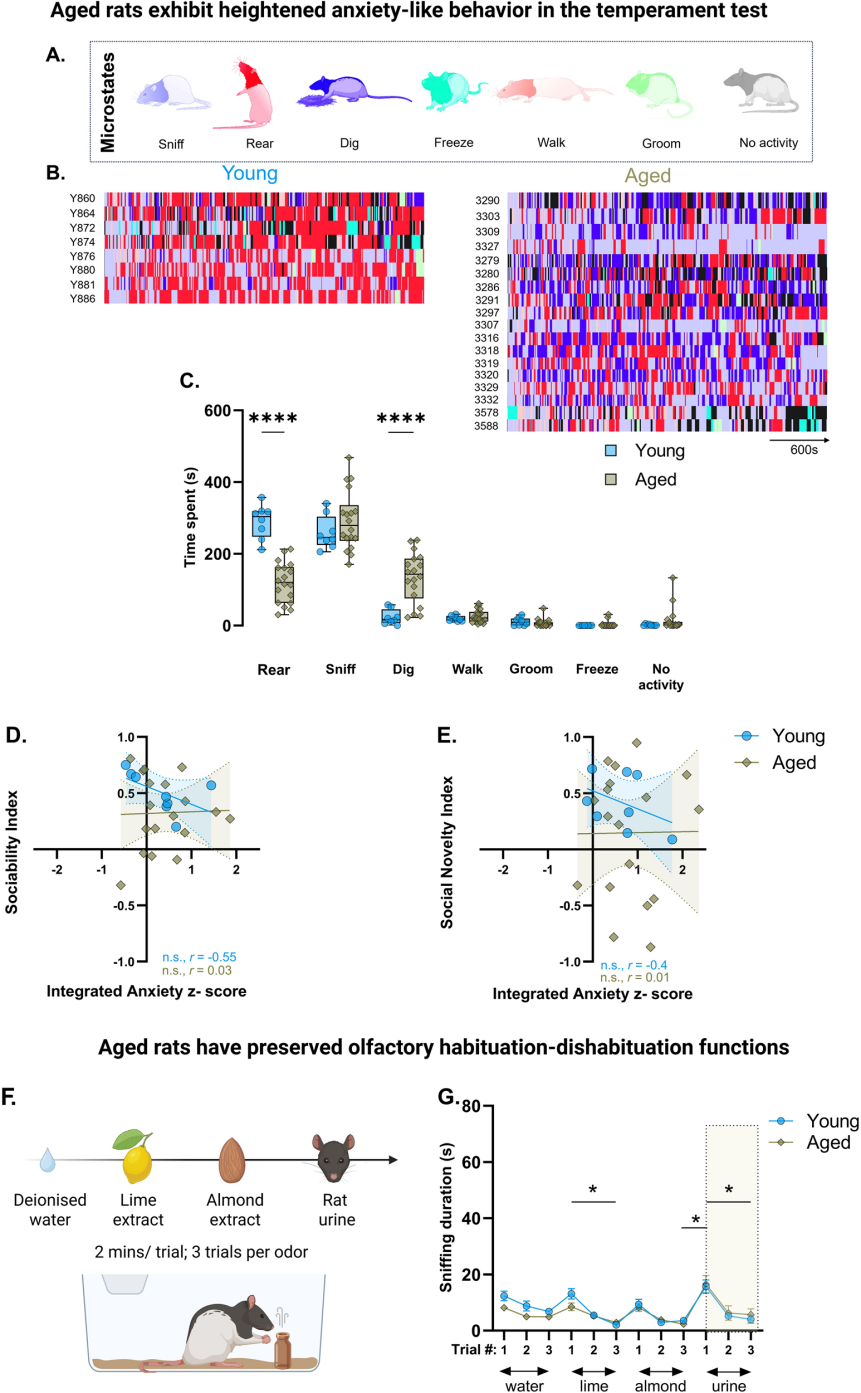
Age-related shift in social familiarity is not a consequence of temperamental changes or sensorimotor processing. ***A***, Seven distinct behaviors (sniffing, rearing, digging, freezing, walking, grooming, and no activity) were identified for quantification. ***B***, Ethograms of individual animals from young and aged groups over 10 min. ***C***, Box and whisker plots with median depicting cumulative time spent in different behavioral states during exploration of a novel environment. Rearing, young = 290.4 ± 16.71 s; aged = 120.3 ± 13.31 s; digging, young = 23.06 ± 7.46 s; aged, 134.1 ± 16.33 s. Two-way ANOVA, main effects of behavior, *F*_(6,168)_ = 144.6; *p* < 0.0001; age × behavior interaction, *F*_(6,168)_ = 20.99; *p* < 0.0001; Bonferroni's post hoc test, ****p < 0.0001 for digging and rearing. *n*: young = 8, aged = 18 rats. ***D***, ***E***, Scatterplots showing the relationship between sociability (Pearson's correlation, *r*, young = −0.55; aged = 0.03) and social novelty (Pearson's correlation, *r*, young = −0.4; aged = 0.01) indices with the integrated anxiety *z*-score derived from the temperament and three-chamber social tests. *n*, young = 8; aged = 18 rats. ***F***, Schematic of the olfaction test. ***G***, Mean olfactory exploration times to different odors (water, lime, almond, and urine) in young and aged rats. Two-way RM ANOVA, main odor effects, *F*_(11, 396)_ = 14.01; *p* < 0.0001; Tukey's post hoc comparisons of habituation and dishabituation between odors: *p < 0.05 for both groups. Data are shown as mean ± SEM. *n*, young = 12 and aged = 26 rats per group.

Next, we asked whether temperament-related characteristics—crucial determinants in shaping cognition, behavior, and emotional responses throughout the lifespan (Kato et al., 2013)—contribute to social novelty bias in aging. We conducted a 10 min assessment to analyze behavioral responses to a novel, otherwise relatively neutral, environment (Fig. 4*A*). Aged rats showed less rearing and increased digging relative to young (Fig. 4*B*,*C*), potentially indicative of anxiety-like traits (de Brouwer et al., 2019). Walking, grooming, and freezing behaviors were comparable between groups (Fig. 4*C*).

Aging is accompanied by changes in an array of additional capacities that might impact three-chamber social exploration, including a decline in motor function. Indeed, aged rats traveled less distance compared with young controls during the habituation, sociability, and social novelty trials (Extended Data Fig. 2-2*A*; Table 1) of the three-chamber social test. Nonetheless, total stimulus-directed exploration was comparable between groups on sociability and social novelty tests (Extended Data Fig. 2-2*B*), suggesting that the motivation and ability to explore are preserved. The findings suggest that physical function fails to account for the blunted social novelty and increased social familiarity bias observed in aged rats.

For evaluation in relation to social novelty, we then calculated an integrated anxiety *z*-score using the three behavioral variables affected in the aged rats (i.e., rearing and digging in the temperament test and reduced distance traveled in sociability or social novelty trials). The analysis failed to reveal any significant associations between this score and either sociability (Fig. 4*D*) or social novelty (Fig. 4*E*). These findings suggest that aging effects on anxiety and exploratory drive are unlikely to account for the familiarity bias observed in many aged rats.

Lastly, we wondered if changes in social behavior in aged rats might be secondary to the deficits in olfactory function frequently reported in aging (Rawson et al., 2012). To test that possibility, olfactory habituation–dishabituation curves were determined in response to neutral, nonsocial, and social odorants (Fig. 4*F*). Olfactory exploration habituated across multiple exposures to a repeated odor in both young and aged rats, with no evidence of differences between groups (Fig. 4*G*). In addition, aged rats showed robust dishabituation to new odors similar to young rats, notably including a greater response to the most socially relevant odor tested, i.e., rat urine. These findings indicate that while changes in olfaction are a reliable feature of aging, these effects are not sufficient to significantly impact the olfactory discrimination demands of the spontaneous social behavior procedure examined here.

### iTBS–TMS treatment influenced social novelty preference in the aged rats, depending on their pretreatment status

Our findings indicate that social novelty preference is selectively vulnerable to aging independent of decline in overall motivational drive for social interaction, changes in memory, or effects of aging on a variety of sensorimotor and behavioral response characteristics. The corresponding mechanistic implication is that individual differences in social cognition in aging likely reflect distinct neural network states that might be predicted to respond differently to intervention. We tested that idea here using noninvasive brain stimulation as a probe to ask whether the pretreatment status of memory and social phenotype in aged animals differentially modulates the social response to iTBS–TMS.

**Figure 5.**
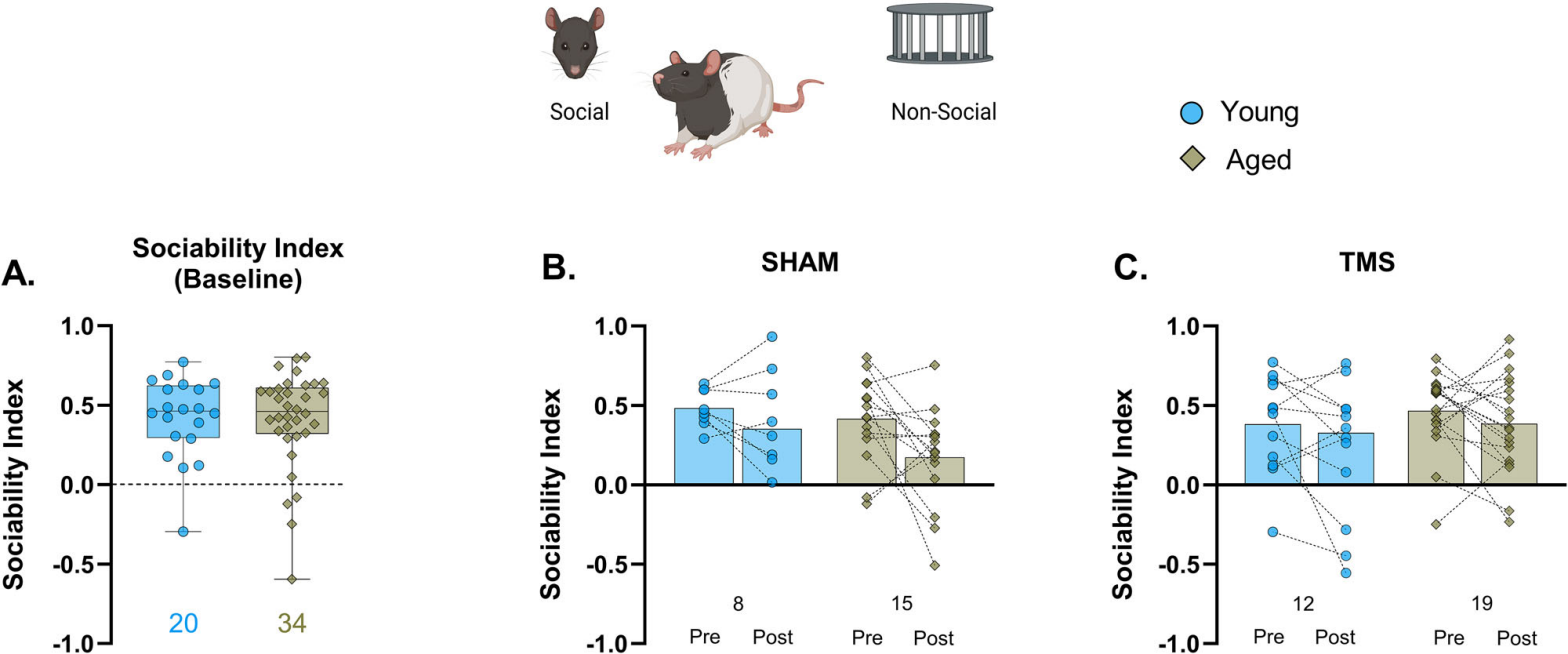
iTBS–TMS treatment did not affect sociability preference. ***A***, Box and whisker plot with median showing the baseline (pretreatment) sociability index scores for young and aged rats. ***B***, ***C***, Mean and individual animal pre- versus posttreatment sociability index scores for sham and TMS young and aged rats. Unpaired (***A***) and paired *t* tests (***B***, ***C***). *n*, young = 20; aged = 34 rats. The sample size for each group is mentioned below their bar graphs. See Extended Data Figure 5-1 depicting the effects of sham and TMS treatment on sociability and social novelty index scores in aged rats subgrouped according to hippocampal memory in the water maze.

**Figure 6.**
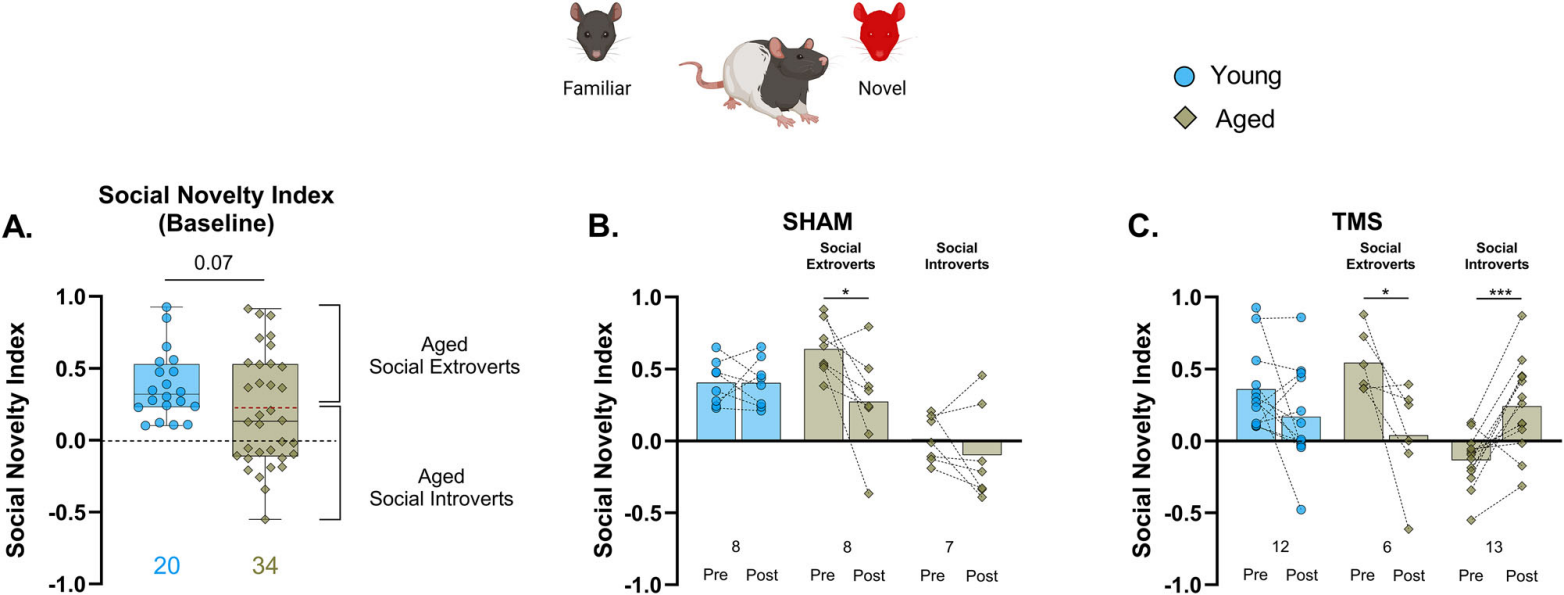
Baseline social phenotype modulates the social novelty response to iTBS–TMS treatment in aged rats. ***A***, Box and whisker plot with median (solid black line) showing the baseline (pretreatment) social novelty index scores in young and aged rats. The red-dotted line in the “aged” group denotes the cutoff value (0.23) for classifying aged social extroverts (score ≥ 0.23) and social introverts (score < 0.23). Unpaired *t* test, *t*_(52)_ = 1.88; *p* = 0.07; young, 0.38; aged, 0.2. ***B***, Mean and individual animal pre- versus posttreatment social novelty index scores for sham conditions in young rats and aged animals subgrouped by baseline social phenotype. Aged social extroverts, paired *t* test, *t*_(7)_ = 2.91; *p = 0.03; pretreatment, 0.64 ± 0.07; posttreatment, 0.27 ± 0.12. Two-way RM ANOVA for Sham treatment, age, *F*_(2, 20)_= 16.17; *p* < 0.0001; treatment, *F*_(1, 20)_ = 7.08; *p* = 0.02; and their interaction, *F*_(2, 20)_ = 3.36; *p* = 0.06. ***C***, Mean and individual animal pre- versus posttreatment social novelty index scores for TMS conditions in young rats and aged animals subgrouped by baseline social phenotype. Aged social extroverts, paired *t* test, *t*_(5)_ = 2.78; *p = 0.04; pretreatment, 0.54 ± 0.09; posttreatment, 0.04 ± 0.15. Aged social introverts, paired *t* test, *t*_(12)_ = 4.39; ***p = 0.0009; pretreatment, −0.13 ± 0.05; posttreatment, 0.24 ± 0.32; effect size, Cohen's *d*, 1.45; 95% CI 0.613–2.44. Two-way RM ANOVA for TMS treatment, age, *F*_(2, 28)_ = 3.61; *p* = 0.04; treatment, *F*_(1, 28)_ = 2.34; *p* = 0.14; and their interaction, *F*_(2, 28)_ = 14.09; *p* < 0.0001.

Replicating our initial observations (Fig. 2*A*), the smaller sample of aged rats available in Experiment 2 showed preserved baseline sociability (Fig. 5*A*), similar to young, and iTBS–TMS had no effect in either age group when sociability was retested 48 h after treatment (Fig. 5*B*,*C*; Extended Data Fig. 5-1*A*,*B*). On the social novelty trial, baseline (pretreatment) social novelty index scores revealed a marked trend toward a social familiarity preference in the aged group, confirming the pattern observed in Experiment 1 (Fig. 6*A*). iTBS had no effect on social novelty in either the young or aged group overall and, moreover, no effect on the aged rats when the results were stratified by memory status, documented in the water maze (Extended Data Fig. 5-1*C*,*D*).

10.1523/ENEURO.0422-25.2025.f5-1 Fig 5-1 **Effects of sham and TMS treatment on sociability and social novelty index scores in aged rats subgrouped according to hippocampal memory in the water maze.** (A, B) Mean and individual animal pre- vs. post-treatment sociability and social novelty index scores in the aged unimpaired and impaired rats. Paired *t*-test for Aged Unimpaired group: *t*_10_ = 2.41, *p = 0.04. Two-way repeated measures ANOVA for sociability: main effects of memory status: *F*_3, 30_ = 1.22; *p* = 0.32, treatment: *F*_1,30_ = 6.14; *p* = 0.02, and their interaction: *F*_3, 30_ = 2.22; *p* = 0.11). For social novelty: main effects of memory status: *F*_3, 30_ = 1.34; *p* = 0.28, treatment: *F*_1,30_ = 0.61; *p* = 0.44, and their interaction: *F*_3, 30_ = 2.38; *p* = 0.09). The sample size for each subgroup is denoted below the respective bars. Download Fig 5-1, TIF file.

However, a different pattern of results emerged when TMS effects on social novelty were examined in the aged animals grouped according to pretreatment social phenotype. A social novelty index cutoff score of 0.23 was derived based on the median split of the 75 aged rats from Experiment 1. Given the relatively large sample size, compared with the alternative option of using cutoffs derived from the smaller sample in Experiment 2, this approach provided a more robust, data-driven estimate of individual variability within the aged group. Aged subjects with a robust social novelty preference of 0.23 and above were operationally classified as “social extroverts,” while those with scores below 0.23, demonstrating either no preference or a familiarity bias, were designated “social introverts” (Fig. 6*A*). Although not evident in the young (Fig. 6*B*), a modest effect of repeated testing was observed primarily in the aged animals, with aged sham rats (Fig. 6*B*) exhibiting a decline in posttreatment social novelty preference scores. A similar pattern was also observed in the aged extroverts receiving TMS (Fig. 6*C*), suggesting that repeated exposure alone fails to enhance a preference for social novelty. Strikingly, in contrast to these patterns, the aged introverts group exhibited an increase in social novelty scores following TMS with a robust effect size (Fig. 6*C*). This directionally opposite change points to a potential neuromodulatory effect of TMS, dependent on social phenotype, rather than a nonspecific influence of repeated testing. Overall, the rat model established here supports the idea that memory and specific features of social cognition are independently vulnerable to aging and that these domain-specific effects arise from distinct neural network states that dictate differential sensitivities to intervention.

## Discussion

Social connectedness powerfully impacts health and well-being in aging, with stronger bonds linked to benefits across a range of physical and behavioral outcomes. The negative influences of loneliness and social isolation are no less potent, including associations with cardiovascular diseases, mood disorders, and dementia (Kennedy and Adolphs, 2012). Defining the direction of causality and specific social capacities affected is challenging in human studies, however, owing partly to the complex nature of social constructs and the many potential confounding variables. As a starting point toward establishing a comprehensive social neuroscience of aging, here we report foundational observations on multiple dimensions of spontaneous social behavior in a rat model, taking advantage of an unusually large subject sample that was well characterized for another prominent feature of cognitive aging, i.e., hippocampal memory.

The findings indicate that different dimensions of social cognition display distinct vulnerabilities to aging, uncoupled from other features of age-related cognitive decline. Notably, sociability—the relative bias for social versus nonsocial stimuli—was intact in aged rats, countering the idea that social engagement deteriorates in old age as a consequence global decline in social motivation (Machanda and Rosati, 2020; Henry et al., 2023; Rothwell et al., 2023). Against this background, aged rats exhibited a reduced preference for novel conspecifics, and as a group, they failed to display the normative tendency to actively explore new social connections. This lack of overall novelty bias, however, is misleading and reflects increased between-subject variability in the older sample, where the distribution of scores greatly exceeded the range for young animals. Strikingly, the distributions were also qualitatively distinct; while novelty preferences in many aged rats rivaled even the strongest bias seen in younger individuals, over a third displayed a social phenotype rarely seen in young controls, comprising a robust bias toward interacting with a familiar conspecific. This age-related shift broadly aligns with humans and NHP observations (Carstensen, 2021; Siracusa et al., 2022), and our findings are among the first to extend this hallmark of increased individual variability to rats.

Identifying potential confounding factors is a significant challenge in developing a preclinical rat model for investigating the social neuroscience of aging (Eldesouky and English, 2019). Compared with young rats, aged animals exhibited reduced exploration, rearing, and increased repetitive digging, i.e., behaviors sometimes indicative of heightened anxiety (Sturman et al., 2018; de Brouwer et al., 2019). Nonetheless, integrated anxiety *z*-scores for these parameters failed to correlate with any of the social indices. We also tested whether olfactory decline contributes to the shift in social familiarity preference observed in older rats, given the role olfaction plays in rodent social interactions (Rennie et al., 2013; Abbas et al., 2024). Young and aged rats showed equivalent olfactory exploration and habituation to novel odors, including the most socially salient stimulus tested (rat urine), which elicited the strongest response in both groups. These findings, together with the observation that sociability is entirely spared, suggest that the effects of aging on temperament and sensorimotor processing are unlikely to account for the pronounced familiarity bias found in a large subgroup of aged rats.

Debate persists about whether social cognition is best understood as a specialized function, reflecting a distinct brain organization, or as an aggregate, emergent property that arises from interactions between other, better-characterized capacities such as memory and attention. By the latter view, it might be that a broad, global decline in cognitive function with age blunts social interaction secondarily rather than age interacting with social cognition processes per se. One possibility along these lines was tested in the current study by evaluating the social behavioral metrics in relation to water maze scores from the same subjects using a standard summary metric of individual differences in hippocampal memory (Haberman et al., 2017; Myrum et al., 2019). The aged rats as a group displayed significant spatial memory impairment, consistent with many earlier reports, but the results from our large sample of 115 animals demonstrate that sociability and social novelty are entirely uncoupled with individual differences in spatial learning and memory in both young and aged rats. These findings provide compelling evidence that the observed effects of aging on social novelty are not simply a proxy for the status of recognition memory but instead reflect a distinct dimension of social cognition.

The working hypothesis that the age-related change in memory and social cognition we report are independent implies that their underlying cause might differ, reflecting at least partly distinct neurobiological conditions. Our earlier study reported the effects of TMS on recognition memory in aged rats vary specifically in relation to individual differences in hippocampal memory, measured in the water maze before TMS intervention (Weiler et al., 2021). Extending that logic, here we directly tested the prediction that the behaviorally dissociable effects of aging on memory and social phenotype would differentially influence the response to high-frequency TMS. Consistent with that idea, while iTBS–TMS had no impact on social indices in young or aged rats overall or when aged rats with or without memory impairment were considered separately, a robust posttreatment shift in social novelty was observed in aged animals distinguished by their social phenotype. Specifically, TMS selectively increased the preference for social novelty in aged social introverts (i.e., rats that preferred a familiar conspecific over a novel one before treatment). In contrast, the bias toward social novelty seen in aged social extroverts declined following either sham or iTBS, presumably reflecting an effect of repeated testing. Thus, the effects of noninvasive brain stimulation on social novelty preference are unrelated to the integrity of hippocampal memory but vary instead in direct relation to individual differences across the full spectrum of social phenotypes among aged rats.

Aging affects the connectivity, composition, and operation of the social decision-making network, i.e., a conserved system of brain regions in humans and rodents consisting of the prefrontal cortex, anterior cingulate, hippocampus, amygdala, and striatum (Lee and Harris, 2013; Hitti and Siegelbaum, 2014; Sugiura et al., 2015; Lee and Williams, 2025). This network is critically involved in making choices in social contexts involving multiple conspecifics (Baez-Mendoza et al., 2021) and is vulnerable to age-related change (Grieve et al., 2005; Raz and Rodrigue, 2006; Maresca et al., 2020), impacting the strategic allocation of neural resources for social decision-making. Whether age-related shifts in oxytocin signaling and excitatory–inhibitory balance in these brain regions contribute to the increased familiarity preference observed in the older rats (Neumann and Slattery, 2016; Lopatina et al., 2018) warrants investigation.

Our findings establish a robust preclinical model for pursuing a detailed social neuroscience of aging, paving the way to test the effects of sex differences, partner loss, and prolonged isolation. It is important to recognize that a brief session of social interaction with a stranger inevitably falls short in matching the depth of familiarity established through enduring human social relationships. Future longitudinal studies that follow paired animals across the lifespan and incorporate complementary social tasks will be essential to define stable social phenotypes and establish cross-paradigm reliability. Additional neurobiological tools also need to be brought to bear, providing greater regional and neurochemical specificity than the relatively untargeted TMS approach used here. The influence of repeated testing observed in some groups also highlights the importance of employing crossover or counterbalanced designs in future studies to better isolate TMS-specific effects from those associated with repeated behavioral exposure. The foundation for pursuing these important directions is now in hand.

## Synthesis

Reviewing Editor: Michael McDannald, Boston College Decisions are customarily a result of the Reviewing Editor and the peer reviewers coming together and discussing their recommendations until a consensus is reached. When revisions are invited, a fact-based synthesis statement explaining their decision and outlining what is needed to prepare a revision will be listed below. The following reviewer(s) agreed to reveal their identity: NONE.

The authors provide a novel assessment of social cognition in Aged rats that is rigorous and powered to uncover important findings. This approach and results should be of considerable interest to neuroscientists focused on aging.

## Author Response

Manuscript title: When Familiar Faces Feel Better: A Framework for Social Neurocognitive Aging in a Rat Model Manuscript #: eN-NWR-0422-25 Response to Comment Minor Comment Page 7, provide the specifics on scoring based on the zones of interest around the stimulus cages in the three-chamber test (e.g., 2 cm toward the stimulus?).

Response:

Investigation of either the social or non-social stimulus was considered when the experimental animal was within ~3 cm of an enclosure with its nose oriented toward the front opening.

These details have been incorporated in the revised manuscript (Please see page no. 7 of the revised article file).
