# Supplementary figures and images for: When Familiar Faces Feel Better: A Framework for Social Neurocognitive Aging in a Rat Model
Source: eNeuro. 2026 Feb 4;13(2):ENEURO.0422-25.2025. doi: 10.1523/ENEURO.0422-25.2025 (PMC12893812; doi:10.1523/ENEURO.0422-25.2025)

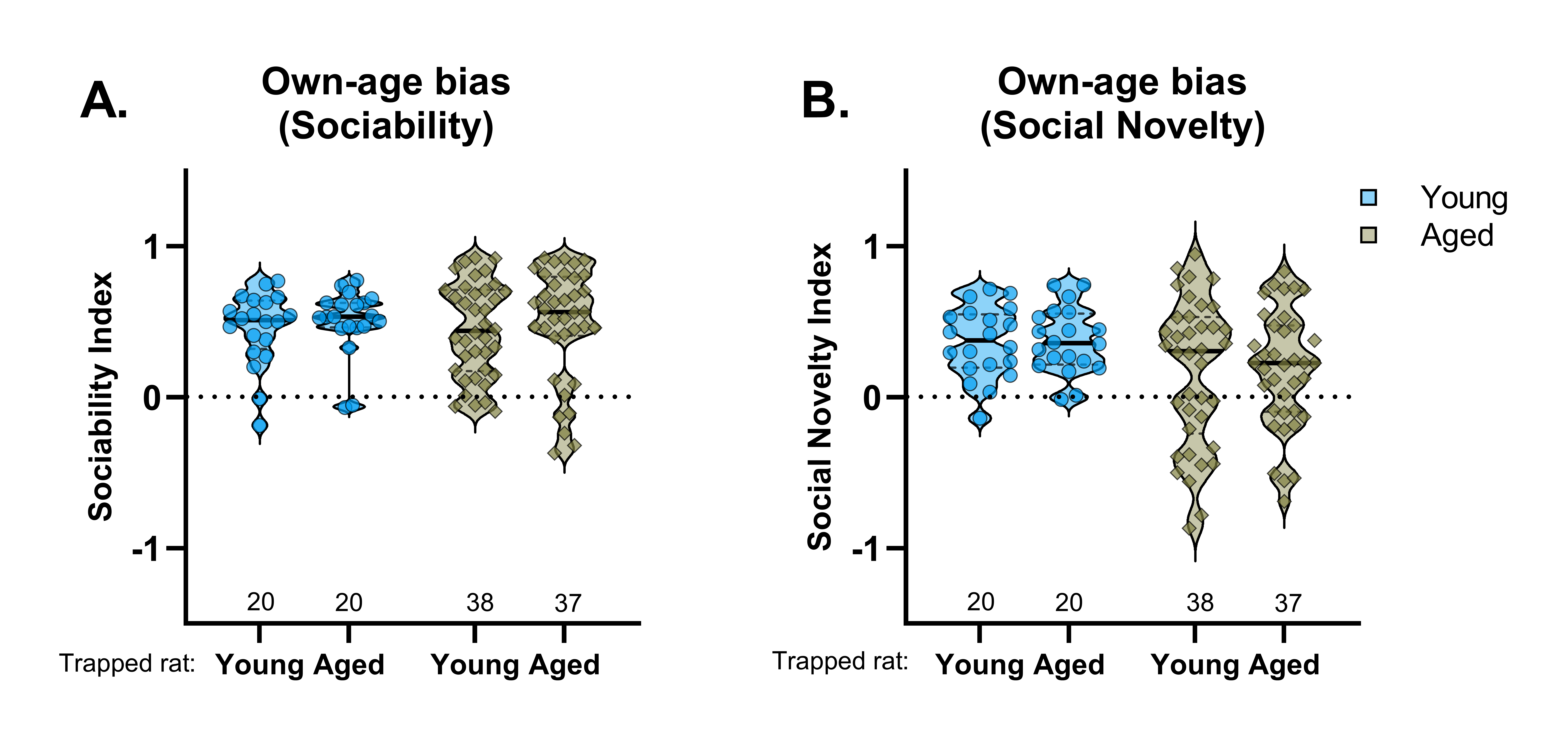

Supplement: Fig 2-1 — Own age bias in young and aged experimental rats. (A) Violin plots with median showing the preference of young and aged rats toward young and aged trapped (stimulus rats) in sociability (A) and social novelty (B) trials. Both age groups showed a similar magnitude of preference for either a young (4-6 months old) or an aged (24-25 months old) trapped conspecific in these trials. The sample size of each condition is depicted below the violin plots. Download Fig 2-1, TIF file. [file eneuro-13-ENEURO.0422-25.2025-s002.tif]

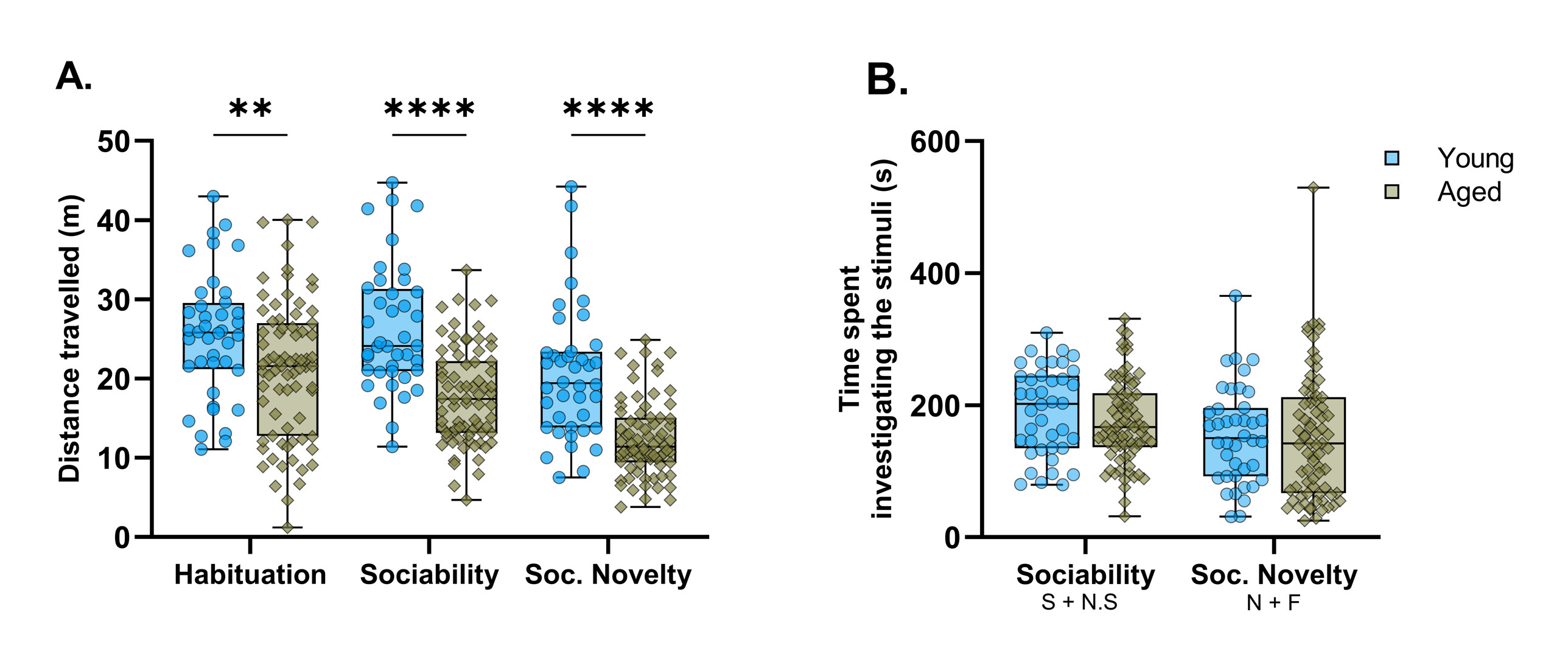

Supplement: Fig 2-2 — Locomotor activity and stimulus-driven exploration of young and aged rats. (A) Box and whisker plots depicting the distance traveled per subject for each phase of testing. Two-way ANOVA: main effect of group: F1,113 = 39.06; p < 0.0001, stimuli or novelty: F2,226 = 46.32; p < 0.0001, and their interaction: F2,226 = 4.89; p = 0.008. Bonferroni’s multiple comparisons test, **p < 0.01, ****p < 0.0001. (B) Box and whisker plots depicting total stimulus-directed exploration time, with individual animal values. S: Social, N.S: Non-social, N: Novel, F: Familiar. Two-way ANOVA: main effects of group: F1,113 = 3.58; p = 0.52, trials: F1,113 = 0.4; p = 0.53, and their interaction: F1,113 = 0.76; p = 0.39. n: young = 40, aged = 75 rats. Download Fig 2-2, TIF file. [file eneuro-13-ENEURO.0422-25.2025-s003.tif]
